# Helping Hands: A Cost-Effectiveness Study of a Humanitarian Hand Surgery Mission

**DOI:** 10.1155/2014/921625

**Published:** 2014-08-20

**Authors:** Kashyap K. Tadisina, Karan Chopra, John Tangredi, J. Grant Thomson, Devinder P. Singh

**Affiliations:** ^1^College of Medicine, University of Illinois at Chicago, Chicago, IL 60612, USA; ^2^Department of Plastic and Reconstructive Surgery, Johns Hopkins University, Baltimore, MD 21287, USA; ^3^Section of Plastic and Reconstructive Surgery, Yale University School of Medicine, New Haven, CT 06511, USA; ^4^Division of Plastic Surgery, University of Maryland Medical Center, Wing S8D, 22 South Greene Street, Baltimore, MD 21201, USA

## Abstract

*Purpose*. Congenital anomalies and injuries of the hand are often undertreated in low-middle income countries (LMICs). Humanitarian missions to LMICs are commonplace, but few exclusively hand surgery missions have been reported and none have attempted to demonstrate their cost-effectiveness. We present the first study evaluating the cost-effectiveness of a humanitarian hand surgery mission to Honduras as a method of reducing the global burden of surgically treatable disease. *Methods*. Data were collected from a hand surgery mission to San Pedro Sula, Honduras. Costs were estimated for local and volunteer services. The total burden of disease averted from patients receiving surgical reconstruction was derived using the previously described disability-adjusted life years (DALYs) system. *Results*. After adjusting for likelihood of disability associated with the diagnosis and likelihood of the surgery's success, DALYs averted totaled 104.6. The total cost for the mission was $45,779 (USD). The cost per DALY averted was calculated to be $437.80 (USD), which is significantly below the accepted threshold of two times the per capita gross national income of Honduras. *Conclusions*. This hand surgery humanitarian mission trip to Honduras was found to be cost-effective. This model and analysis should help in guiding healthcare professionals to organize future plastic surgery humanitarian missions.

## 1. Introduction

Humanitarian missions to low-middle income countries (LMICs) have become a major source of medical care for underserved populations, particularly in plastic surgery. Teams consist of a variety of healthcare professionals who travel to the country in need, with all required supplies and equipment. On location, surgeons perform life-changing procedures for patients with congenital deformities, trauma, or burns, all of which cause significant disease burden on the local population [1, 2]. This service is provided free of charge to the patients. All expenses are paid by charitable donations, usually without religious, financial, cultural, or political agendas [3].

Honduras is a democratic nation in Central America with a population of approximately 7.5 million. Over half of the population lives below the poverty line and an estimated 30% are unemployed [4]. Like many LMICs, Honduras lacks both resources and an adequate health care infrastructure to provide the care for its citizens. According to the World Health Organization, “roughly 30.1% of the population receives no healthcare, 83% are uninsured, and there is marked exclusion of ethnic minorities and rural populations.” Further, there are only 8.8 physicians and 3 nurses per 10,000 citizens, compared to 26 physicians and 94 nurses per 10,000 in the United States [5]. In 2005, the per capita total expenditure on healthcare in Honduras was $91 versus $6,350 in the United States [4]. Furthermore, patients' access to hospitals can be limited geographically and by a lack of transportation means. At the same time, medical technology and surgical techniques in developed countries continue to advance rapidly. This has created a growing dichotomy in healthcare between rich and poor countries [6, 7]. In particular, areas of subspecialty surgery, such as reconstructive plastic surgery and hand surgery, which are increasingly specialized in countries like the United States, are all but absent in some developing nations [8, 9]. Because of this, humanitarian missions to the developing world are becoming more and more relevant as a way to provide direct aid and training to local surgeons. Many medical and surgical mission trips throughout the world have been reported in the literature with plastic surgery volunteer trips being especially common, and the majority of which involve cleft lip and palate repair [10]. Some reported mission trips involve treatment of both hand anomalies and craniofacial defects. However, very few of the reported humanitarian mission trips have focused solely on hand surgery. Further, the cost-effectiveness of such trips has not been previously reported. In the present paper, we present a report on the cost-effectiveness of a mission trip to Honduras in May 2006 that exclusively focused on hand surgery.

## 2. Methods

### 2.1. Study Population

In May 2006, our group of 20 healthcare professionals traveled to San Pedro Sula, Honduras. Our local sponsor, the Ruth Paz Foundation, a nonprofit charitable group, assisted on site with organization, logistics, and advertising. We worked out of a local public hospital called Leonardo Martinez, which hosts a variety of medical and surgical humanitarian mission trips. Team personnel consisted of 3 hand/microsurgery trained surgeons, 1 plastic surgery trained surgeon, 1 hand/microsurgery fellow, 1 plastic surgery resident, 3 anesthesiologists, 1 pediatrician, 1 nurse anesthetist, 5 operating room nurses, 1 recovery room nurse, 1 hand therapist, 1 team administrator, and 1 photographer. All surgical supplies (including gowns, drapes, sponges, sutures, dressings, and plaster) and surgical instruments were brought with the team for the trip.

Members of the Ruth Paz Foundation set up the screening clinic and organized the follow-up visits. Potential patients were alerted about the available services, through radio announcements and fliers. The majority of the patients were screened for surgery on the primary screening day with additional patients, who missed the main screening day, screened each day. The operating schedule for the next five days was created based on the patients seen on the main screening day. Those screened for surgery were then immediately referred to waiting anesthesiologists and pediatrician for same day medical clearance. Patients were then instructed when to return for surgery before they left.

Many minor procedures were performed with local surgeons present in order to provide training for their future practice. Ganglion cysts and masses were removed for extreme size, intractable pain, or functional limitation. Because of the team's yearly trip to Honduras, we were also able to perform more complex two-staged procedures. All surgeries were performed by either a board certified plastic surgeon or orthopedic surgeon. Each day, the team would round on all postoperative patients in the morning and in the evening. The patients were seen in follow-up clinic by local physicians, who removed splints, dressings, sutures, and k-wires, as necessary. Patients were also seen by local physical and occupational therapists that provided assistance with splints as well as therapy.

### 2.2. Costs

The team's costs for the trip were calculated by adding the team's travel expenses, which included transportation, lodging, and donated supplies that were brought with them (Table 1(a)). The team's 2006 costs were then adjusted for inflation to present day based on data obtained from World Bank's data [4]. Weekly hospital personnel salaries and preoperative, intraoperative, and postoperative medication costs were obtained from the Ruth Paz Foundation (Tables 1(b) and 1(c)). We were unable to obtain operative room cost or daily hospital stay costs. Other fixed costs, such as utilities and building costs, were not included as we were not able to obtain this information. Patients were charged a symbolic fee based on their household income by the local hospital for services, but due to the nominal nature of the fee (ranging from $0 to $50), this was not included for analysis.

### 2.3. Outcome

The total burden of musculoskeletal disease was calculated for each patient that underwent surgery using disability-adjusted life years (DALYs) format. As no surgery performed was life-saving, all of the DALYs attributed were from years lost to disability (YLD) and none from years of life lost (YLL). YLD is calculated using disability weight and the remaining life expectancy. In previous calculations of YLD, age weighting factors and discount rate were also incorporated in the calculation; however, the recently published Global Burden of Disease 2010 study has moved away from those adjustments [11]. Every patient's diagnosis and associated disability was matched as closely as possible to a health state based on each state's lay description as described in Global Burden of Disease 2010 study. Each patient was then assigned a disability weight based on the closest available health state (Table 2). For each patient, the potential years lived with disability value was calculated using the patient's age and life expectancy chart found in the Global Burden of Disease 2010 study. For each patient, the DALY value represents the burden of an untreated condition. This value has been subsequently adjusted for likelihood of permanent disability and likelihood of treatment success as described in the literature by McCord and Chowdry and modified by Gosselin et al. [12–16] and represents the DALYs averted with surgery (Table 3). To err on side of overestimating cost per DALY averted, we chose conservative weights for disability, likelihood of permanent disability, and effectiveness of treatment. The scoring system used in assigning likelihood of permanent disability and likelihood of treatment success is shown in Table 4.

## 3. Results

In total, 120 patients were screened and 80 patients were found to be candidates for surgery. Over the week, 128 total procedures were performed on 54 adults (68%) and 26 children (32%). The average age of the patient undergoing surgery was 31 years with ages ranging from 10 months to 68 years. Of these patients, 27 were female (34%) and 53 were male (66%). Table 4 includes the procedures performed on each patient, as well as their age and gender. Operative time for the entire trip totaled 93 hours and 50 minutes over 5 days. Average operative time was 18 hours and 46 minutes per day and 6 hours and 15 minutes per operative table per day. Most of the procedures were very short in duration, with 43 cases (53%) lasting less than 1 hour. 25 cases (31%) took 1-2 hours, 9 cases (11%) lasted 2-3 hours, and only 4 cases (5%) were longer than 3 hours in duration. No immediate complications, such as ischemic loss or early wound infection, were noted. There were no anesthetic complications and no mortalities.

As shown in Table 4, the total number of DALYs potentially avertable totaled 220.5. Adjusting for likelihood of disability associated with the diagnosis and likelihood of the surgery's success, DALYs averted totaled 104.6. The total cost (in current USD) for the volunteer trip including the team's travel and lodging cost of $45,779.18 and local hospital's cost of $2,903 (USD) is detailed in Table 1. On average, it costs $572.24 (USD) per patient that was surgically treated. Cost-effectiveness was measured using cost per DALY averted and, for this trip, the cost for each DALY averted was conservatively estimated to be $437.80 (USD), which is significantly less than the accepted threshold of two times the per capita gross national income of Honduras, $3,890 (USD). Further, a brief sensitivity analysis provided in Table 1(e) displays that even if total costs were to increase by 500%, the cost per DALY averted would still be below the threshold of $3,890 (USD).

## 4. Discussion

This study demonstrates that hand surgery mission trips are a cost-effective means of providing surgical care at HNQCP in San Pedro Sula, Honduras, using an established economic evaluation model. We also inherently validate the effectiveness of the DALY system as a useful and versatile method of evaluating surgical mission trips. While it is one of the first quantitative systems of evaluating such trips, it is also only one of the many possible ways to analyze mission trips. However, this analysis also represents an important step in standardizing the evaluation of such trips to better optimize foreign intervention by surgical teams, as proposed by McCord [13].

The $437.80 per DALY averted for this week long surgical mission trip is similar to those previously reported in the literature that have ranged between $343 and $362 per DALY [14, 15]. Our cost per DALY is well within the two times per capita gross national income, an accepted metric for program cost-effectiveness as suggested by earlier studies [15]. We believe our slightly higher cost per DALY averted can be attributed to multiple factors. First, we have used conservative estimations for all DALY and disability weight. Second, previous studies did not use the 2010 version of the Global Burden of Disease (GBD) system to evaluate cost-effectiveness and consequently may have contributed to differences in the cost per DALY averted value. Third, bringing more staff, such as residents, anesthesiologists/anesthetist, pediatrician, nurses, and hand therapist, may have added to travel and lodging cost. As the availability of more locally trained medical professionals is available in Honduras, fewer anesthesiologists, or nurses, and therapists from the United States will be needed for each trip thus making each subsequent mission trip more cost-effective than the previous. With local capacity to care for simple cases like ganglion cyst removal, trigger finger release, and arthrodesis, subsequent trips can focus on more disabling complex conditions such as nerve injuries which require advance surgical training. Since a volunteer mission trip's costs are relatively fixed, focusing on these conditions can contribute to more DALYs averted lowering the trip's cost per DALY averted.

In addition to providing direct care, our team has been able to lecture at the medical school in San Pedro Sula and invite local surgeons to come and learn how to manage surgical hand cases. Our nurses and therapists have also worked with local staff to improve pre-, peri-, and postoperative care of patients. Such training and educational efforts are often difficult to quantify and are not reflected in the cost per DALY averted, but they are important in the long-term development of adequately trained local health care professionals and healthcare infrastructure. While the capacity to care for these surgical conditions is being developed in Honduras, surgical mission trips such as ours serve as an important bridge until that day arrives.

The limitations of this study include the inability to include certain costs, such as operating room, hospital stay, utilities, and building costs; however, given the large margin between the cost per DALY averted and the twice per capita gross national income (PCGNI) of Honduras ($3890 in 2012) [4], we believe that the underreported costs have only a minor impact on the cost per DALY averted. Even if total costs (TC) were five times higher, the cost per DALY averted would still be the threshold value of $3890 (2∗PCGNI), as illustrated in Table 1(e). As in previous cost-effectiveness studies, a rough trade-off is used to assign hand conditions with disability weights as the Global Burden of Disease 2010 study does not have many specific disability weights for various hand conditions. There are also instances where disability weights make little sense from a functional standpoint: amputation of finger(s) excluding thumb has a disability weight of 0.030 which compares poorly to disability weight of 0.013 for amputation of thumb, long term. One can argue that the loss of a thumb is more functionally debilitating than the loss of a finger as opposition-apposition function is lost in a thumb amputation and grip maybe minimally affected with a finger amputation [11]. The nature of short volunteer mission trip makes obtaining long-term outcomes data difficult. However, with greater capacity in host countries, prospective studies that assess patient outcomes will enable us to more objectively determine patient outcomes without relying on assumptions. Until then, we feel that using the correctional factor “probability of successful treatment” is needed to account for treatment success/failures as there is a lack of follow-up data. Imperfect as it maybe, the DALY method for assessing cost-effectiveness has been used in a number of previous studies in LMICs and offers a more objective and standardized way to assess the impact of surgical mission trips, cost-effectiveness and serves as a benchmark for future trips.

## Figures and Tables

**Table:** (a) Team costs

	Value (USD) in 2006	Cumulative inflation rate (2006 to 2013)	Inflation adjusted value (USD) in 2013	% of total costs
Transportation and lodging	$23,000	15.90%	$26,650.34	62.2%
Donated supplies	$14,000	15.90%	$16,226.00	37.8%
Total cost	**$37,000**	**15.90%**	**$42,876.34**	

**Table:** (b) Local personnel salary

	Weekly salary (USD) in 2013	Number	Weekly cost (USD)	% of total costs
Local surgeon	$345	2	$690	45.3%
Surgical tech.	$185	2	$370	24.3%
Nurse	$185	2	$370	24.3%
Cleaning	$92	1	$92	6.0%
Total cost			**$1,522**	

**Table:** (c) Hospital costs

	Weekly cost (USD) in 2013
Pre- and postoperative medications	$448.84
Intraoperative medications	$932
Total costs	**$1,380.84 **

**Table:** (d) Overall mission cost

	Cost (USD)	% of total costs
Team costs	$42,876.34	93.7%
Local personnel costs	$1,522.00	3.3%
Hospital costs	$1,380.84	3.0%
Total overall costs	**$45,779.18 **	

**Table:** (e) Cost-effectiveness metrics

		If 2 × TC (i.e., doubled)	If 5 × TC
Total cost (TC)	$45,779.18	**$91,558.36**	**$228,895.90**
Cost per patient	$572.24	$1,144.48	$2,861.20
Cost per DALY averted	$437.80	$875.32	$2,188.30∗
			∗Still below $3,890 (2 × PCGNI)

*PCGNI: per capita gross national income.

**Table 2:** Patient characteristics.

Age	Sex	Diagnosis	Available disability weight	Disability weight
9	F	Tendon adhesion	Musculoskeletal problems: arms, mild	0.024
16	M	Finger flexor tendon injury	Musculoskeletal problems: arms, mild	0.024
26	M	Finger flexor tendon injury and nerve laceration	Injured nerves: long term	0.136
60	M	Posttraumatic joint contracture	Musculoskeletal problems: arms, mild	0.024
68	F	Trigger finger	Musculoskeletal problems: arms, mild	0.024
3	F	Finger flexor tendon injury	Musculoskeletal problems: arms, mild	0.024
13	M	Cubitus varus	Disfigurement: level 1	0.013
14	M	Polydactyly	Disfigurement: level 1	0.013
47	M	Lipoma	Disfigurement: level 1, with itch or pain	0.029
57	F	Trigger finger	Musculoskeletal problems: arms, mild	0.024
11	M	Burn scar contracture	Burns of <20% total surface area or <10% total surface area if head or neck or hands or wrist involved: long term, with or without treatment	0.018
12	M	Burn scar contracture	Musculoskeletal problems: arms, mild	0.024
21	F	Partial traumatic amputation	Amputation of finger(s), excluding thumb: long term, with treatment	0.03
30	F	Nerve laceration	Injured nerves: long term	0.136
34	M	Skin contracture	Musculoskeletal problems: arms, mild	0.024
47	M	Radius and ulna fracture	Fracture of radius or ulna: short term, with or without treatment	0.065
8	M	Metacarpal fracture	Musculoskeletal problems: arms, mild	0.024
11	M	Cubitus varus	Disfigurement: level 1	0.013
21	M	Burn scar contracture	Burns of <20% total surface area or <10% total surface area if head or neck or hands or wrist involved: long term, with or without treatment	0.018
45	M	Posttraumatic joint contracture	Musculoskeletal problems: arms, mild	0.024
48	M	Carpal tunnel syndrome	Injured nerves: short term	0.065
51	F	Ganglion cyst	Disfigurement: level 1, with itch or pain	0.029
67	M	Dupuytren's contracture	Musculoskeletal problems: arms, moderate	0.114
5	M	Burn scar contracture	Burns of <20% total surface area or <10% total surface area if head or neck or hands or wrist involved: long term, with or without treatment	0.018
7	M	Polydactyly	Disfigurement: level 1	0.013
19	F	Tumor	Disfigurement: level 1, with itch or pain	0.029
21	M	Tumor	Disfigurement: level 1, with itch or pain	0.029
31	M	Dorsal ganglion cyst	Disfigurement: level 1, with itch or pain	0.029
42	F	Nonunion radius	Fracture of radius or ulna: long term, without treatment	0.05
56	F	De Quervain's syndrome	Musculoskeletal problems: arms, moderate	0.114
11 mo	M	Thumb hypoplasia	Amputation of thumb: long term	0.013
23	M	Burn scar contracture	Musculoskeletal problems: arms, mild	0.024
33	M	Burn scar contracture	Musculoskeletal problems: arms, moderate	0.114
37	F	Hook nail deformity	Disfigurement: level 1	0.013
48	M	Dupuytren's contracture	Musculoskeletal problems: arms, mild	0.024
8	M	Flexor tendon injury	Musculoskeletal problems: arms, mild	0.024
10	F	Scar contracture	Musculoskeletal problems: arms, mild	0.024
20	M	Flexor tendon injury with nerve laceration	Injured nerves: long term	0.136
55	M	Carpal tunnel syndrome	Injured nerve: short term	0.065
5	M	Syndactyly	Musculoskeletal problems: arms, mild	0.024
17	M	Thumb hypoplasia	Musculoskeletal problems: arms, moderate	0.114
18	M	Foreign body with ulnar neuropathy	Injured nerves: short term	0.065
23	M	Foreign body	Musculoskeletal problems: arms, mild	0.024
34	M	Radial head fracture	Fracture of radius or ulna: short term, with or without treatment	0.065
44	M	Posttraumatic joint contracture	Musculoskeletal problems: arms, mild	0.024
49	M	Extensor tendon laceration	Musculoskeletal problems: arms, mild	0.024
20	M	Flexor tendon injury	Musculoskeletal problems: arms, moderate	0.114
22	F	Posttraumatic joint contracture	Musculoskeletal problems: arms, mild	0.024
22	M	Posttraumatic joint contracture	Musculoskeletal problems: arms, mild	0.024
47	M	Shoulder lipoma	Disfigurement: level 1, with itch or pain	0.029
61	F	Ganglion cyst and ulnocarpal abutment	Musculoskeletal problems: arms, moderate	0.114
5	M	Burn scar contracture	Burns of <20% total surface area or <10% total surface area if head or neck or hands or wrist involved: long term, with or without treatment	0.018
32	M	Flexor tendon injury	Musculoskeletal problems: arms, moderate	0.114
60	F	Ganglion cyst	Disfigurement: level 1, with itch or pain	0.029
61	M	Finger flexor tendon injury	Musculoskeletal problems: arms, moderate	0.114
65	F	L ulna nonunion	Fracture of radius or ulna: long term, without treatment	0.05
10	M	Syndactyly	Musculoskeletal problems: arms, mild	0.024
24	M	Malunion	Musculoskeletal problems: arms, moderate	0.114
35	F	Nerve laceration	Injured nerves: long term	0.136
38	M	Nerve laceration	Injured nerves: long term	0.136
10 mo	M	L MF-RF syndactyly	Musculoskeletal problems: arms, mild	0.024
17	M	Malunion	Fracture of hand: long term, without treatment	0.016
18	M	Flexor tendon injury	Musculoskeletal problems: arms, moderate	0.114
28	M	Radial nerve laceration	Injured nerves: long term	0.136
42	F	Carpal tunnel syndrome	Injured nerve: short term	0.065
14	M	Flexor tendon injury with nerve laceration	Musculoskeletal problems: arms, moderate	0.114
23	F	Burn scar contracture	Burns of <20% total surface area or <10% total surface area if head or neck or hands or wrist involved: long term, with or without treatment	0.018
50	M	Radial nerve laceration	Injured nerves: long term	0.136
9	F	Burn scar contracture	Burns of <20% total surface area or <10% total surface area if head or neck or hands or wrist involved: long term, with or without treatment	0.018
16	F	Malunion	Musculoskeletal problems: arms, mild	0.024
31	F	Ganglion cyst	Disfigurement: level 1, with itch or pain	0.029
47	F	Ulnocarpal abutment	Musculoskeletal problems: arms, moderate	0.114
55	F	Carpal tunnel syndrome	Injured nerve: short term	0.065
14	F	Burn scar contracture	Musculoskeletal problems: arms, moderate	0.114
39	F	Ganglion cyst	Disfigurement: level 1, with itch or pain	0.029
42	M	Posttraumatic joint contracture	Musculoskeletal problems: arms, mild	0.024
46	M	Posttraumatic joint contracture	Musculoskeletal problems: arms, mild	0.024
57	F	Carpal tunnel syndrome	Injured nerve: short term	0.065
23	M	Finger mass	Disfigurement: level 1, with itch or pain	0.029
62	M	Skin lesion	Disfigurement: level 1, with itch or pain	0.029

**Table 3:** DALY averted.

Case #	Age	Remaining life expectancy	Sex	Diagnosis	Procedure	Disability Wt.	DALY	Likelihood of permanent disability	Likelihood of treatment success	DALY averted
Day 1										
1	9	77.27	F	Tendon adhesion	L wrist exploration w/tenolysis FDP	0.024	1.85448	0.7	0.7	0.9086952
2	16	70.3	M	Finger flexor tendon injury	R index finger Hunter rod placement	0.024	1.6872	0.7	0.7	0.826728
3	26	60.41	M	Finger flexor tendon injury and nerve laceration	R FPL repair w/tendon grafts; nerve repair with sural nerve graft	0.136	8.21576	0.7	0.7	4.0257224
4	60	27.81	M	Posttraumatic joint contracture	R long finger PIP joint arthrodesis	0.024	0.66744	0.7	0.7	0.3270456
5	68	20.68	F	Trigger finger	R LF trigger finger release	0.024	0.49632	0.7	0.7	0.2431968
6	3	83.23	F	Finger flexor tendon injury	L ring finger FDS/FDP-Hunter rod implant	0.024	1.99752	0.7	0.7	0.9787848
7	13	73.29	M	Cubitus varus	L lateral closing wedge osteotomy of supracondylar for cubitus varus	0.013	0.95277	0.7	0.7	0.4668573
8	14	72.29	M	Polydactyly	B/l thumb partial duplication repair; anlage excision	0.013	0.93977	0.7	0.7	0.4604873
9	47	39.9	M	Lipoma	Excision of L forearm mass	0.029	1.1571	0.7	0.7	0.566979
10	57	30.55	F	Trigger finger	R IF and LF trigger finger release	0.024	0.7332	0.7	0.7	0.359268
11	11	75.28	M	Burn scar contracture	L forearm excision of burn scar; STSG	0.018	1.35504	0.3	0.7	0.2845584
12	12	74.28	M	Burn scar contracture	R hand thumb webs space deepening with split thickness skin graft	0.024	1.78272	0.3	0.7	0.3743712
13	21	65.36	F	Partial traumatic amputation	L RF amputation completion	0.03	1.9608	0.7	0.7	0.960792
14	30	56.46	F	Nerve laceration	Nerve graft L ulnar nerve; anticlaw tendon transfer	0.136	7.67856	0.7	0.7	3.7624944
15	34	52.52	M	Skin contracture	L middle PIP contracture release and skin graft	0.024	1.26048	0.7	0.7	0.6176352
16	47	39.9	M	Radius and ulna fracture	ORIF L radius/ulna	0.065	2.5935	0.3	0.7	0.544635

Day 2										
17	8	78.26	M	Metacarpal fracture	R LF pinning of metacarpal fracture	0.024	1.87824	0.7	0.7	0.9203376
18	11	75.28	M	Cubitus varus	R supracondylar osteotomy	0.013	0.97864	0.7	0.7	0.4795336
19	21	65.36	M	Burn scar contracture	R IF burn contracture release; FTSG	0.018	1.17648	0.7	0.7	0.5764752
20	45	41.8	M	Posttraumatic joint contracture	L index and long finger PIP fusion	0.024	1.0032	0.7	0.7	0.491568
21	48	38.95	M	Carpal tunnel syndrome	L carpal tunnel release	0.065	2.53175	0.7	0.7	1.2405575
22	51	36.12	F	Ganglion cyst	Excision of R wrist mass	0.029	1.04748	0.7	0.7	0.5132652
23	67	21.55	M	Dupuytren's contracture	L hand excision of Dupuytren's contracture	0.114	2.4567	0.7	0.7	1.203783
24	5	81.25	M	Burn scar contracture	R hand burn contracture release	0.018	1.4625	0.7	0.7	0.716625
25	7	79.26	M	Polydactyly	Reconstruction of R thumb polydactyly	0.013	1.03038	0.7	0.7	0.5048862
26	19	67.34	F	Tumor	Excision of L hand mass	0.029	1.95286	0.7	0.7	0.9569014
27	21	65.36	M	Tumor	Excision of bony tumor ×2 of L humerus	0.029	1.89544	0.7	0.7	0.9287656
28	31	55.48	M	Dorsal ganglion cyst	Excision of ganglion cyst	0.029	1.60892	0.7	0.7	0.7883708
29	42	44.71	F	Nonunion radius	Repair nonunion radius	0.05	2.2355	0.7	0.7	1.095395
30	56	31.47	F	De Quervain's syndrome	De Quervain's release	0.114	3.58758	0.7	0.7	1.7579142
31	11 mo	85.21	M	Thumb hypoplasia	R thumb amp, and pollicization	0.013	1.10773	0.7	0.7	0.5427877
32	23	63.38	M	Burn scar contracture	PIP arthrodesis; debulk flap	0.024	1.52112	0.7	0.7	0.7453488
33	33	53.5	M	Burn scar contracture	Contracture release of all fingers R hand; flexor tendon division	0.114	6.099	0.7	0.7	2.98851
34	37	49.58	F	Hook nail deformity	V-Y advancement L index fingertip	0.013	0.64454	0.7	0.7	0.3158246
35	48	38.95	M	Dupuytren's contracture	L little finger arthrodesis and k-wire for palmar scar revision w/FTSG	0.024	0.9348	0.7	0.7	0.458052

Day 3										
36	8	78.26	M	Flexor tendon injury	2nd stage flexor tendon reconstruction; removal hunter rod and tendon graft from leg to finger	0.024	1.87824	0.7	0.7	0.9203376
37	10	76.27	F	Scar contracture	L hand scar revision; tenolysis; removal of k-wire	0.024	1.83048	0.7	0.7	0.8969352
38	20	66.35	M	Flexor tendon injury with nerve laceration	Repair flexor tendons wrist with tendon grafts, ulnar nerve repair with sural nerve graft	0.136	9.0236	0.7	0.7	4.421564
39	55	32.38	M	Carpal tunnel syndrome	R carpal tunnel release	0.065	2.1047	0.7	0.7	1.031303
40	5	81.25	M	Syndactyly	Syndactyly release L 4th web space	0.024	1.95	0.7	0.7	0.9555
41	17	69.32	M	Thumb hypoplasia	R thumb opponensplasty; R 1st web deepening; R thumb UCL reconstruction	0.114	7.90248	0.7	0.7	3.8722152
42	18	68.33	M	Foreign body with ulnar neuropathy	Excision of foreign body L hypothenar eminence; neurolysis ulnar nerve	0.065	4.44145	0.7	0.7	2.1763105
43	23	63.38	M	Foreign body	Bullet removal ×2 R hand	0.024	1.52112	0.7	0.7	0.7453488
44	34	52.52	M	Radial head fracture	L radial head excision	0.065	3.4138	0.7	0.7	1.672762
45	44	42.77	M	Posttraumatic joint contracture	L LF, RF, and SF PIP joint fusion	0.024	1.02648	0.7	0.7	0.5029752
46	49	38	M	Extensor tendon laceration	Tendon transfer for thumb extension PL → EPL	0.024	0.912	0.7	0.7	0.44688
47	20	66.35	M	Flexor tendon injury	Zone II IF, MF, and RF hunter rods	0.114	7.5639	0.7	0.7	3.706311
48	22	64.37	F	Posttraumatic joint contracture	L LF PIP joint arthrodesis	0.024	1.54488	0.7	0.7	0.7569912
49	22	64.37	M	Posttraumatic joint contracture	L thumb IP fusion	0.024	1.54488	0.7	0.7	0.7569912
50	47	39.9	M	Shoulder lipoma	Excision of R shoulder lipoma	0.029	1.1571	0.7	0.7	0.566979
51	61	26.91	F	Ganglion cyst and ulnocarpal abutment	R dorsal ganglion/L matched ulnar arthroplasty	0.114	3.06774	0.7	0.7	1.5031926

Day 4										
52	5	81.25	M	Burn scar contracture	R hand burn scar contracture release; FTSG	0.018	1.4625	0.7	0.7	0.716625
53	32	54.49	M	Flexor tendon injury	R FDS → FDP transfer 2–5	0.114	6.21186	0.7	0.7	3.0438114
54	60	27.81	F	Ganglion cyst	L dorsal wrist excision of ganglion cyst	0.029	0.80649	0.7	0.7	0.3951801
55	61	26.91	M	Finger flexor tendon injury	L wrist ECRL to FDP; transfer of w/palmaris graft	0.114	3.06774	0.7	0.7	1.5031926
56	65	23.29	F	L ulna nonunion	ORIF with iliac crest bone graft	0.05	1.1645	0.7	0.7	0.570605
57	10	76.27	M	Syndactyly	Release syndactyly 2nd and 4th web spaces with flaps and grafts	0.024	1.83048	0.7	0.7	0.8969352
58	24	62.39	M	Malunion	R thumb MCP joint arthrodesis	0.114	7.11246	0.7	0.7	3.4851054
59	35	51.53	F	Nerve laceration	Tendon transfer for L wrist extension and thumb extension; FCU → ECRB, PL → EPL	0.136	7.00808	0.7	0.7	3.4339592
60	38	48.6	M	Nerve laceration	R forearm sural nerve graft	0.136	6.6096	0.7	0.3	1.388016
61	10 mo	85.21	M	L MF-RF syndactyly	L MF-RF syndactyly release	0.024	2.04504	0.7	0.7	1.0020696
62	17	69.32	M	Malunion	Thumb osteotomy and alignment-ORIF; removal of foreign body thumb	0.016	1.10912	0.7	0.7	0.5434688
63	18	68.33	M	Flexor tendon injury	L forearm FDS → FDP tendon transfer	0.114	7.78962	0.7	0.7	3.8169138
64	28	58.44	M	Radial nerve laceration	Tendon transfer radial nerve palsy; FCR → EDC; PT → ECRB; ring sublimis → EPL	0.136	7.94784	0.7	0.7	3.8944416
65	42	44.71	F	Carpal tunnel syndrome	L carpal tunnel release	0.065	2.90615	0.7	0.7	1.4240135

Day 5										
66	14	72.29	M	Flexor tendon injury with nerve laceration	R FDS/FDP ring and small finger tenorrhaphy and digital nerve repair	0.114	8.24106	0.7	0.7	4.0381194
67	23	63.38	F	Burn scar contracture	L hand burn scar contracture release	0.018	1.14084	0.7	0.7	0.5590116
68	50	37.05	M	Radial nerve laceration	Tendon transfer of radial nerve palsy; FCR → EDC; PT → ECRB; ring sublimis → EPL	0.136	5.0388	0.7	0.7	2.469012
69	9	77.27	F	Burn scar contracture	Web space deepening and scar revision	0.018	1.39086	0.7	0.7	0.6815214
70	16	70.3	F	Malunion	R RF MCP arthrodesis	0.024	1.6872	0.7	0.7	0.826728
71	31	55.48	F	Ganglion cyst	Excision of R wrist dorsal ganglion	0.029	1.60892	0.7	0.7	0.7883708
72	47	39.9	F	Ulnocarpal abutment	L ulnar shortening	0.114	4.5486	0.7	0.7	2.228814
73	55	32.38	F	Carpal tunnel syndrome	R carpal tunnel release	0.065	2.1047	0.7	0.7	1.031303
74	14	72.29	F	Burn scar contracture	L LF PIP burn contracture release and fusion; FTSG; Z plasty L elbow burn scar	0.114	8.24106	0.7	0.7	4.0381194
75	39	47.62	F	Ganglion cyst	Excision of R volar wrist ganglion cyst	0.029	1.38098	0.7	0.7	0.6766802
76	42	44.71	M	Posttraumatic joint contracture	L thumb MCP arthrodesis	0.024	1.07304	0.7	0.7	0.5257896
77	46	40.85	M	Posttraumatic joint contracture	L long finger PIP joint arthrodesis	0.024	0.9804	0.7	0.7	0.480396
78	57	30.55	F	Carpal tunnel syndrome	L carpal tunnel release	0.065	1.98575	0.7	0.7	0.9730175
79	23	63.38	M	Finger mass	Excision of L ring finger mass	0.029	1.83802	0.7	0.7	0.9006298
80	62	26	M	Skin lesion	Excision of L hand skin lesion	0.029	0.754	0.7	0.7	0.36946

Mean:	30.759	55.938				Total:	220.009		Total:	104.349028

R = right; L = left; IF= index finger; LF = long finger; RF = ring finger; SF = small finger; PIP = proximal interphalangeal; MCP = metacarpal phalangeal; FPL = flexor pollicis longs; FDS = flexor digitorum superficialis; FDP = flexor digitorum profundus; FCR = flexor carpi radials; FCU = flexor carpi ulnaris; PL = palmaris longus; EPL = extensor pollicis longus; ECRL = extensor carpi radialis longus; ECRB = extensor; carpi radialis brevis; EDC = extensor digitorum communis; FCU = flexor carpi ulnaris; UCL = ulnar collateral ligament; STSG = split thickness skin graft; FTSG = full thickness skin graft; ORIF = open reduction internal fixation.

**Table 4:** Scoring system.

	Weight
Likelihood of permanent disability	
>95% go on to disability	1.0
<95 and >50%	0.7
<50 and >5%	0.3
<5%	0
Effectiveness of treatment	
>95% chance for cure	1.0
<95 and >50%	0.7
<50 and >5%	0.3
<5%	0
